# Adlay, an ancient functional plant with nutritional quality, improves human health

**DOI:** 10.3389/fnut.2022.1019375

**Published:** 2022-12-22

**Authors:** Wen F. Weng, Yan Peng, Xin Pan, Jun Yan, Xiang D. Li, Zhi Y. Liao, Jian P. Cheng, An J. Gao, Xin Yao, Jing J. Ruan, Mei L. Zhou

**Affiliations:** ^1^College of Agriculture, Guizhou University, Guiyang, Guizhou, China; ^2^Key Laboratory of Coarse Cereal Processing in Ministry of Agriculture and Rural Affairs, School of Food and Biological Engineering, Chengdu University, Chengdu, Sichuan, China; ^3^Southwest Guizhou Institute of Karst Regional Development, Xingyi, Guizhou, China; ^4^College of Life and Environmental Sciences, Wenzhou University, Wenzhou, China; ^5^Institute of Crop Sciences, Chinese Academy of Agricultural Sciences, Beijing, China

**Keywords:** adlay, functional ingredient, medicinal value, nutritional effect, biotechnology

## Abstract

Adlay (*Coix lacryma-jobi* L.), a crop closed related to maize (*Zea mays* L.) and sorghum (*Sorghum bicolor* L.), originated in tropical/subtropical regions of Asia and Africa; southwest China primary center of this plant’s origin, evolution and migration. Adlay is a traditional high-value minor crop used for both medicinal and dietary purposes. Adlay has anti-tumor, anti-bacterial, anti-inflammatory, analgesic, blood sugar-lowering, and blood lipid-lowering effects. To clarify the main bioactive components and phytochemical compounds and to fully explore their utility, this review summarizes the research done on the main functional ingredients of adlay, including amino acids and proteins, oils, vitamins and minerals, polysaccharides, and polyphenols. This study also highlighted the application of genome sequencing to tailor nutrient-rich adlay cultivars and nutraceutical product development. Additionally, the acquisition of high-density genomic data combined with next-generation phenotypic analysis will undoubtedly improve our understanding of the potential genetic regulation of adlay nutraceutical traits. This review provides new insights and ideas for the research of adlay in comparison and evolutionary genomics, and a useful reference for molecular breeding and genetic improvement of this important minor crop.

## 1 Introduction

**FIGURE 1:**
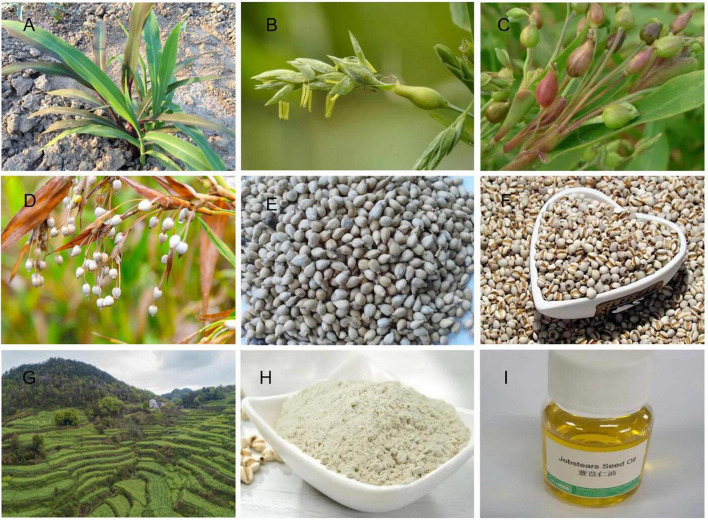
Adlay (*Coix lacryma-jobi* L.) of Southeast Asia. **(A)** Seedling, **(B)** inflorescence, **(C)** immature shelled seeds, **(D)** ripe hulled seeds, **(E)** threshed hulled seeds, **(F)** threshed seeds, **(G)** field, **(H)** adlay seed flour, **(I)** coix seed oil. Photograph by Anjing Gao.

Adlay (*Coix lacryma-jobi* L.) is a uniquely shaped and colorful heirloom grain that originated in Asia and Africa. Adlay is named in this way because of its teardrop shape, and it is one of the few non-hybrid grains available at present. The main edible and medicinal part of adlay is the seed kernel. It is a green food with high protein, medium fat, and low sugar content. When the fruits mature in the autumn, the plants are harvested, air-dried, and the fruits are then sun-dried to remove the outer husk, yellow-brown seed coat, and impurities, and to collect the seed kernels. White coix seed kernels are wide, or oblong, 4_8 mm long and 3_6 mm wide. The surface is milky white and smooth, with an occasional yellowish _brown seed coat. One end is blunt and round, and the other end is wider and slightly concave with a light brown punctate hilum. The back is round and convex, and there is a wide and deep longitudinal groove on the ventral surface (Figure 1). Its grains provide a creamy, sweet flavor and chewy texture, which is delightful for salads, pilafs, soups, and sides.

**FIGURE 2:**
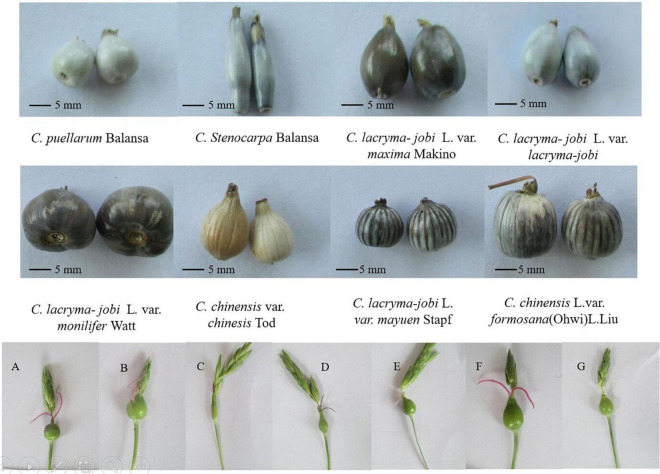
Plant morphology, inflorescence and involucre of adlay germplasm accessions. **(A–G)** Different young fruits and stigmas. Photograph by XL.

Adlay, an ancient cereal crop, is distributed in India, China, Japan, Korea, Malaysia, Vietnam, and Indonesia, among other regions. Guizhou, Guangxi, Yunnan, and other areas in southwestern and southern China are part of the origin of adlay. The unique geographical environment and climatic conditions of China have created abundant adlay resources (1). Guizhou, due to the “three-dimensional climate” and complex topographical conditions in the mountainous areas, has a diverse ecological environment, and the coix germplasm resources are rich. The color of mature adlay husk varies greatly, with a total of six colors: white, yellow_white, gray, brown, dark brown, and black. Wild adlay grains were encapsulated in a black stony hull and cultivated in a white papery hull. The black stony hull is thick and difficult to dehull, whereas the white papery hull is thin and can be easily removed (Figure 2). China was one of the first countries to use coix seed as a health food, and it has also achieved many results in research on the functional components of coix seed (2). However, at present, the development of functional ingredients from adlay crops is still mainly concentrated on the seeds, and the use of active ingredients from other plant parts is relatively limited (3). A large number of non-seed adlay parts cannot be fully utilized, inevitably leading to low profitability in the adlay industry, which in turn restricts the further development and research on the functional components of adlay (4).

It has high nutritional value and pharmacological effects, including antioxidant, anti-inflammatory, anti-cancer, anti-tumor, hypoglycemic, and analgesic effects (5). The ratio of protein to carbohydrate was relatively high; the content of potassium, iron, calcium and fiber was relatively high; and the content of fat was relatively low. Fermentation and sprouting are effective ways to improve the nutritional value and biological activity of adlay (6–8). Coix seeds can be cooked, roasted, pureed and mixed with breadcrumbs and herbs. It can be shaped into miniature cakes for use as a delicious and nutritious appetizer. In recent years, with the improvement in people’s quality of life, research on functional food ingredients has received increasing attention (9). Research on the chemical composition of adlay began in the 1960s (10). To date, researchers have isolated more than 70 compounds, including lipids, sterols, phenols, from adlay (11, 12). As for the research on the functional components of adlay, early emphasis was placed on the determination and analysis of common nutrient components, such as proteins, amino acids and vitamins in its seeds (13). However, in recent years, the focus has shifted to functional research into the utilization of adlay chemical components, particularly the mining of functional components and pharmacological testing of plant non-seed parts (14). To make full use of the adlay’s resources, it is necessary to fully study its nutritional and medical function components and to clarify utilization forms to maximally exploit their functional roles (2). Here, we summarize the progress made in the studies on adlay origin and germplasm resources, bioactive compounds and healthy benefits, and genome and germplasm resource genetic improvement. We also suggest future development directions to provide a reference for studying metabolic mechanisms and molecular breeding of adlay and further promote its development as a functional health food.

## 2 The origin and germplasm resources of adlay

### 2.1 The origin of adlay

Adlay originated in the tropical or subtropical regions of Southeast Asia and was one of the most important crops in ancient times and even during the Xia-Shang period in China (15). Southwest China is one of the primary centers of the origin, evolution and migration of adlay (16). Adlay is an ancient crop and one of the earliest gramineous plants developed and utilized in China (1). According to documentary records and archaeological findings, adlay has a history of ∼8,000 years of cultivation and domestication in China. As early as the Xia Dynasty to the Spring and Autumn Period and the Warring States Period, the collection and planting of coix has received widespread attention and has become an important food crop (17). China has a history of more than 2,500 years of planting adlay, with a relatively wide range of cultivation, and has accumulated rich experience in adlay planting and field management. Adlay has a wide distribution area, almost all over the north and south, but it is mainly distributed in subtropical regions such as Sichuan, Yunnan, and Guizhou, and it generally grows in wet areas such as ditches, rivers, and fields. Huang et al. (18) confirmed that Guangxi, Hainan, Guizhou, and Yunnan are the primary centers of adlay in China based on the number of coix species and the distribution density of wild species, while the middle and lower reaches of the Yangtze River and Northern provinces (regions) were secondary centers formed by the gradual northward movement, domestication, and selection of coix. Through chromosome detection, it was found that the aquatic coix seed in the southwest of Guangxi had the original base number of chromosomes (2*n* = 10) i.e., the original diploid, whereas in other areas of China, the chromosome types of coix seed were all tetraploid (2*n* = 20) germplasms. Therefore, southwestern Guangxi is one of the origins of coix plants (19). The planting area and output of adlay in Guizhou Province ranks first in China, and it has become the largest processing cluster and product distribution center of adlay in China and neighboring countries.

### 2.2 The germplasm resources of adlay

South Asia, Southeast Asia and their neighboring regions are the regions with the most abundant germplasm resources of adlay in the world. Coix is a short-day crop, but the distribution of germplasm resources is very wide, from low-latitude Southeast Asia to China’s Liaoning, and even South Korea, and Japan. Pandey et al. (20) analyzed 54 accessions of adlay germplasm resources in India. Four groups can be distinguished by the character, size, color, and texture of the involucre, and they believe that northeastern India is a major diversity center of adlay. China has one of the richest types of adlay germplasm resources worldwide. There are five species and four varieties of adlay germplasm in China (21). Coix included five species of *C. aquatica, C. chinensis*, *C. lacryma-jobi*, *C. puellarum Balansa*, and *C. stenocarpa* (Table 1). Chinese wild coix seedlings were covered with white powdery wax on the back of the flag leaves, especially the stalk epidermis, which can reduce transpiration and prevent water from entering the stems when flooded (Figure 1). This is one of the reasons why it can tolerate waterlogging and drought. Wild coix is a cross-pollinated perennial short-day plant. Under natural conditions, after inter-plant or intra-plant pollination in the wild population, the seeds mature, fall to the ground in autumn, and germinate into new plants in the following spring (22). Simultaneously, new tillers grow at the base of old stems and develop into fertile branches. Directed breeding to improve and utilize wild genetic resources to achieve target traits has become a breeding goal to accelerate the accumulation of good genes and cultivate new adlay varieties (23). The inheritance of color, texture, beak and longitudinal stripes of coix involucre is controlled by one to two pairs of alleles and characterized by gene interaction, gene overlap, and partial segregation inheritance, which can be used as important marker traits for coix genetic improvement (19). Adlay resources contain huge pharmaceutical development value and have broad application prospects. If these resources can be fully developed and utilized and their added value greatly increases, this will not only optimize resource utilization but also promote the large-scale development of the adlay industry (24). The development of basic disciplines, such as the mining and utilization of coix germplasm resources and analysis of genetic basis, has become an important driving force for the improvement of traditional breeding levels (25). Adlay has strong stress resistance, high barren tolerance, and tolerance to extensive cultivation conditions. The demand for environmentally friendly crops and a more diverse food supply for humans and animals provide new opportunities to grow adlay in the drier and warmer environmental conditions predicted in the future. However, towing to the lack of excellent varieties, the yield is low, which severely limits the development of adlay. After years of investigation and collection of adlay germplasm resources, more than 500 resources were collected. Because of various project developments, the destruction of the native resources of wild and aquatic wild adlay resources is also quite serious. Therefore, protection of adlay resources is necessary and urgent.

**TABLE 1:** Coix genera classification.

Classification	Species and variant names	References
1 species and 1 variant	*Coix agrestis*	Encyclopedia of Chinese Agriculture
	*C. lacryma-jobi* var. *frumentacea*	Encyclopedia of Chinese Agriculture
3 species and 4 variants	*C. lacryma-jobi*	
	*C. lacryma-jobi* var. *lacryma-jobi*	(78)
	*C. lacryma-jobi* var. *monilifer*	(46)
	*C. lacryma-jobi* var. *mayuen*	(21)
	*C. lacryma-jobi* L. var. *formosana*	(77)
	*C. pushlarum*	(86)
	*C. stenocarpa*	
4 species and 8 variants	*C. lacryma-jobi*	
	*C. lacryma-jobi* var. *lacryma-jobi*	
	*C. lacryma-jobi* var. *perlarium*	
	*C. lacryma-jobi* var. *inflatum*	(1)
	*C. lacryma-jobi* var. *monilifer*	(4)
	*C. lacryma-jobi* var. *strobialaceum*	(20)
	*C. lacryma-jobi* var. *compressum*	(61)
	*C. lacryma-jobi* var. *mayuen*	(86)
	*C. lacryma-jobi* L. var. *formosana*	Encyclopedia of Chinese
	*C. pushlarum*	Agriculture
	*C. stenocarpa*	
	*C. aquatic* Roxb	
5 species and 4 variants	*C. aquatica* Roxb	
	*C. pushlarum* Balansa	
	*C. chinensis.* Tod	
	*C. chinensis.* var. *chinesis* Tod	(16)
	*C. chinensis.* var. *formosana* (Ohwi) L.	(4)
	Liu	(17)
	*C. lacryma-jobi* L	(11)
	*C. lacryma-jobi* var. *Lacryma-jobi*	(34)
	*C. lacryma-jobi* var. *maxima* Makino	
	*C. stenocarpa* Balansa	

## 3 Bioactive compounds and health benefits of adlay

### 3.1 Proteins

The protein content of adlay is remarkably high, especially in the seed kernels, which contain about 20% protein, with some reports of wild-type coix seed containing 31.72% protein content. The coix shell also contains 2.17_2.80% protein. The structure and function of coix seed proteins have always been a focus of research. The quality of coix seed protein is excellent, with gliadin, gluten, globulin, and albumin accounting for 44.74, 37.38, 6.20, and 1.43% of the total protein, respectively. The main components of coix seed protein are gliadin and gluten, which account for 82.12% of the total protein. Gliadin was separated by using sodium dodecyl sulfate-polyacrylamide gel electrophoresis (SDS-PAGE). Gliadins can be divided into α (19 and 22 kD), β (14 kD), γ (16 and 27 kD), and δ (10 kD) according to their molecular weights (26). Comparison of the amino acid sequences of the coix seed protein revealed that the amino acid sequence homology of corn, sorghum and coix seed proteins was more than 50%. Coix seed protein has anti-inflammatory activity, and its mechanism of action may be to regulate the activation of the IKK/NF-κB signaling pathway and control the production and secretion of inflammatory factors. Li et al. (27) confirmed that coix seed gliadin-derived small molecule peptide (CPP) has a significant promoting effect on the proliferation of mouse splenic lymphocytes and shows a dose-effect relationship. Liu et al. (28) isolated an antifungal protein with significant activity against *Trichoderma viride* from adlay seeds, use a combination of chromatographic focusing and ion-exchange chromatography. Luo et al. (29) used gel filtration chromatography, ion exchange chromatography, and hydrophobic chromatography to quickly separate antifungal proteins in coix seeds and obtained four proteins. Two of the proteins strongly inhibit the growth of *T. viride*, *Poplar canker*, and *Fusarium graminearum*, and they are chitosanases with a high degree of homology. Ruan et al. (22) isolated an antifungal protein with significant activity against *Mycosphaerella melonis*, *Helminthosporium turcicum*, *Alternaria solani*, *Phytophthora capsici*, *Isariopsis griseola*, and *Colletotrichum gloeosporioides* from the coixs seeds by combining aqueous two-phase aqueous extraction, affinity chromatography, and centrifugal ultrafiltration.

Coix seed contains 18 amino acids, eight of which are essential. Coix seeds are rich in glutamate, proline, and leucine, and lysine is the limiting amino acid. The ratio of essential amino acids is closed to that required by the human body, and with some functional amino acids also being present. Functional amino acids refer to amino acids that have special functions in addition to protein synthesis, and mainly include glutamic acid, alanine, branched chain amino acids, tryptophan, sulfur-containing amino acids, and glycine. Both kernels and non-kernels adlay are rich in amino acids (28). The total amount of amino acids in coix seed kernels was as high as 19.72%, which is 2.2 times that in rice kernels. Among them, 6.27% are essential amino acids with a balanced amino acid composition (the average essential amino acid index value was 58.97). Coix seeds contain many types of functional amino acids, with glutamic acid accounting for 25.85%, and branched-chain amino acids (leucine, and valine) for as much as 23.5%; antioxidant amino acids (tyrosine, cysteine, methionine, and tryptophan) account for 10.8%, and the contents of lysine and tryptophan are higher than those in rice, maize and wheat (9). The non-seed part of the adlay also contains 18 amino acids, including seven essential amino acids. The total amount of amino acids and the seven essential amino acids in the endothelium were the highest at, 11.34_13.98 and 3.55_4.26%, respectively. Followed by the roots and exoculum, the total amount of amino acids and the contents of 7 essential amino acids were 4.26_6.75 and 1.37_1.68%, 1.53_6.85 and 0.51_1.75%, respectively (28).

### 3.2 Adlay polysaccharide (Coixan)

Coixan is an important basic component for the pharmacological activity of coix seeds (30). Coix seeds contain a variety of active polysaccharides, including polysaccharides A, B, and C, neutral dextran 1-7, acid polysaccharides CA-1 and CA-2, and many other ingredients, mainly composed of rhamnose, arabinose, mannose, galactose, dextran, and other monosaccharides, with a molecular weight of approximately 1.5 × 10^4^ (31, 32). Among them, polysaccharide A is composed of rhamnose, arabinose, xylose, mannose, and galactose in a ratio of 1:1:1:11:10; polysaccharide B is composed of rhamnose, arabinose, xylose, mannose, galactose, and glucose in a ratio of 3:18:13:3:10:5; and polysaccharide C is a type of dextran (33). Coixan is a water-soluble, acid-neutral polysaccharide, pH 6.8, which has good heat stability and does not contain nucleic acids or proteins (34, 35). Hong et al. (31) used the phenol-sulfuric acid method to determine the content and quality of polysaccharides from non-seed plant parts (roots, stems, and leaves) and stems of different origins and harvest periods. The polysaccharides content in the roots, stems, and leaves differed slightly and were stable at higher levels (roots 54.374 mg/g, stems 56.9322 mg/g, and leaves 52.7549 mg/g). From June to November, the content of polysaccharides in the stems of adlay showed an increasing trend, and the polysaccharide content of the stem in different producing areas varied widely (26).

Coixan effectively kills and inhibits the growth of cancer cells, significantly lowers blood sugar levels, and improves immune function (18). Coixan is physiologically active; it is a multi-effect free radical and metal ion inhibitor and has a strong anti-cancer effect (36, 37). According to a previous study, coixan can reduce the blood sugar of diabetic mice caused by tetraoxopyrimidine i.e., coixan has the ability to reduce blood sugar, probably by the inhibit decomposition of liver glycogen, glycolysis of muscle glycogen and gluconeogenesis. Coix seed polysaccharides alleviate type 2 diabetes in mice by activating IGF1/PI3K/AKT signaling through gut microbiota- derived short-chain fatty acids (38). Coixan can significantly improve the abnormal morphology of rat T lymphocytes and the abnormal immune adhesion performance of rat red blood cells. Coixan is targeted and widely use in the treatment of malignant tumors; it can also better control the inflammatory response, regulate the abnormal immune function of the body, reduce the pain caused by malignant tumors, and avoid long-term drug treatment causing tumor resistance (36). Coixan can stimulate the immune system by activating upstream immune cells such as dendritic cells (DC), thereby promoting cell proliferation and cytokine release of T lymphocytes, leading to tumor cell death. Coixan also has the effect of reducing drug toxicity and increasing drug efficacy. Coixan can increase the content of short-chain fatty acids in the cecum and plays an anti-complement and immune-regulating role by affecting the activity of macrophages. Liu et al. (39) found that coixan can reduce the serum leptin content in rats while increasing the serum adiponectin content, so that coixan can significantly reduce body weight, improve physical signs, and reduce fasting blood glucose and 2 h postprandial blood glucose levels. Kang and Song (40) injected tetraoxopyrine-treated diabetic mice and adrenaline hyperglycemic mice intraperitoneally with 50 and 100 mg/kg coixan, respectively, to reduce blood glucose levels. The mechanism underlying blood glucose reduction is coixan’s inhibition of the decomposition of liver glycogen and glycolysis of muscle glycogen, thereby inhibiting the gluconeogenesis and reducing blood sugar; it also prevented or treated the occurrence of vascular complications of diabetes. Kim et al. (41) found that coixan can significantly improve immunity in mice and promote lymphocyte transformation while exhibiting activities related to reducing blood sugar and regulating immunity. Manosroi et al. (42) proved that coixan is a good proton (H^+^) donor that scavenges free radicals and inhibit lipid peroxidation; it can scavenge a variety of free radicals and chelate metals. Coixan can significantly enhance the antioxidant function in mice, inhibit the reduction of spleen and thymus indices in immunocompromised mice, enhance the macrophage phagocytic index and lymphocyte proliferation response, restore the immune function of immunosuppressed mouse models, increase the serum half_hemolysis value (HC_50_), and effectively alleviate the effects of carbon tetrachloride (CCI4)-induced hepato toxicity (42). Coixan can protect pancreatic β cells and have therapeutic effects on various types of diabetes. Further research on the clinical treatment of diabetes with coixans is warranted.

### 3.3 Coicis oil, coixenolide and coixol

Liu et al. (2) used thin-layer chromatography and gas chromatography-mass spectrometry (GC-MS) to separate and identify coix seed lipids and determined that the neutral part of the coix seed lipid is mainly composed of triglycerides (>85%), followed by glycerin monoesters, diglycerides and fatty acid hydrocarbon esters. Functional lipids are fats with special physiological functions that are both beneficial and medicinal. They mainly include polyunsaturated fatty acids, phospholipids, and structural greases (18). Adlay seeds have high fat content and are rich in functional lipids. Among them, the highest content of fatty acids is in bran, up to 36%; followed by adlay seed kernels, with a fat content of up to 7%, which is 5.8 times that in rice (43). Fatty acids are also present in the shells of seed kernels. Adlay fat consists of over 83% unsaturated fatty acids (oleic and linoleic acid), which are beneficial to the humans’ body (Table 2). This also includes palmitic acid, myristic acid, coix acid, coix ester, fat-soluble hormone, stearic acid, and other functional lipids (44). This type of fat has high value for human health.

**TABLE 2:** Total phenolic content, ORAC values, PSC values, EC_50_ of cellular antioxidant activities and CAA values of different fractions derived from defatted Job’s tears seed meal, comparisons and compound standards (mean ± SD, *n* = 3).

Extract and comparisons^a^	TPC (mg GAE/g)	ORAC (μmol TE/g)	PSC (μmol TE/g)	CAA (μmol QE/g)	EC_50_ (μg/ml)	Cytotoxicity^b^ (mg/ml)
CAE	22.96 ± 1.66b^c^	695.25 ± 15.14^b^	147.03 ± 8.31^b^^c^	29.80 ± 1.11^b^^c^	157.88 ± 2.26^e^	>5
BF	43.84 ± 2.94^c^	717.65 ± 33.88^b^	185.59 ± 15.92^c^	41.38 ± 0.55^c^	113.70 ± 3.98^d^	>5
AF	9.8 ± 0.83^a^	138.47 ± 15.87^a^	64.41 ± 6.50^a^^b^	19.55 ± 1.01^a^^b^	244.12 ± 3.11^f^	>5
SF1	6.34 ± 0.61^a^	88.45 ± 11.50^a^	35.00 ± 3.76^a^	10.67 ± 0.59^a^	441.10 ± 5.57^g^	>5
SF2	134.60 ± 5.06^d^	1932.67 ± 62.07^c^	1037.88 ± 28.56^d^	97.66 ± 9.02^d^	48.342.55^c^	1.23 ± 0.16
SF3	183.42 ± 3.27^e^	2500.76 ± 65.10^e^	1454.45 ± 72.55^e^	153.57 ± 8.51^e^	30.68 ± 1.46^b^	>5
BHT	–	–	60.56 ± 3.73^a^^b^	–	–	–
Vc	–	2071.78 ± 53.45^d^	2705.09 ± 140.79^f^	196.21 ± 14.95^f^	24.02 ± 0.82^a^	0.87 ± 0.09

**Table:** 

Compound standards	ORAC (μmol TE/g)	PSC (μmol TE/g)	CAA (μmol QE/g)	EC_50_ (μg/ml)	Cytotoxicity^b^ (mg/ml)
Vanillic acid	5972.42 ± 137.70^c^	565.39 ± 13.04^a^	156.816.06^a^	30.01 ± 0.74^d^	>0.1
Ferulic acid	5683.99 ± 119.02^c^	2367.95 ± 111.21^b^	259.21 ± 12.69^a^	18.17 ± 0.83^c^	>0.1
Rutin	2658.83 ± 242.30^a^	2199.71 ± 126.55^b^	1143.88 ± 80.98^b^	4.12 ± 0.11^b^	>0.1
Quercetin	4615.05 ± 116.51^b^	5010.43 ± 61.79^c^	3313.69 ± 146.70^c^	1.42 ± 0.06^a^	>0.1
Vanillin	6552.47 ± 214.80^d^	No activity	No activity	No activity	>0.1
p-Coumaric acid	6975.22 ± 210.36^d^	No activity	No activity	No activity	>0.1

Bars with no letters in common are significantly different (*p* < 0.05). TPC, total phenolic content; ORAC, oxygen radical absorbance capacity; PSC, peroxyl radical scavenging capacity; CAA, cellular antioxidant activity; EC_50_, concentration for 50% of maximal effect; CAE, crude acetone extract; BF, n-butanol fraction; AF, aqueous fraction; SF1, sub-fraction 1; SF2, sub-fraction 2; SF3, sub-fraction 3; Vc, ascorbic acid; BHT, butylated hydroxytoluene.

^a^Comparisons are butylated hydroxytoluene (BHT) and ascorbic acid (Vc).

^b^Concentration at which the absorbance decreased by more than 10% when compared to the control was considered to be toxic.

^c^Values with different letters are significantly different (*p* < 0.05) (70, 86).

**FIGURE 3:**
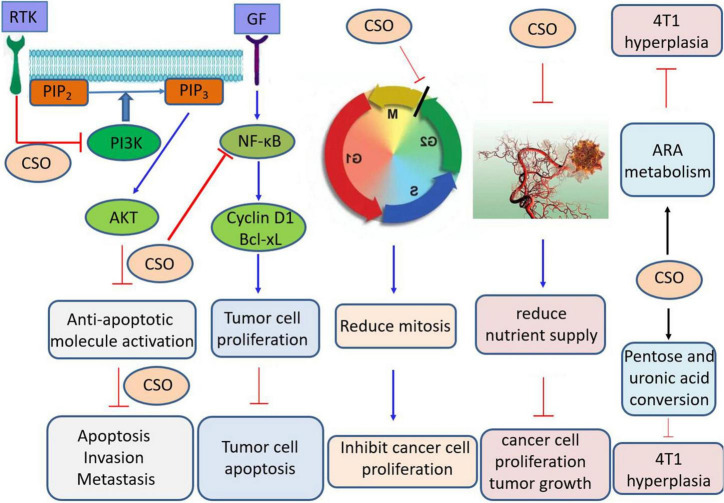
Schematic diagram of physiological mechanisms of coix seed oil. CSO, Coix seed oil; AKT, Serine/threonine kinase; ARA, Arachidonic acid; GF, Growth factor; NF-kB, Nuclear factor-kappa B; PIP2, phosphati-dylinositol-4,5-bisphosphate; PIP3, Phosphatidylinositol 3,4,5, triphosphate; PI3K, Phosphatidylinositol 3-kinase; RTK, Receptor tyrosine kinase; Bcl-XL, B-Cell Leukemia/Lymphoma XL.

Coicis oil (Kanglaite in a clinical setting) is a fatty oil extracted from adlay seeds. Coicis oil contains coixenolide, palmitic acid, stearic acid, octadecenoic acid, myristic acid, and palmitate (45, 46). Coix seed volatile oil contains 69 components, the main of which are hexanal, hexanoic acid, 2-ethyl-3-hydroxy-hexylbutrate, γ-non-aldactone, nonanoic acid, octanoic acid, ethylpalmitate, methyllinoleate, vanillin, and ethyl linoleate (47). At low concentrations, coicis oil has a stimulatory effect on breathing, and on cardiac striated and smooth muscles, which can significantly dilate pulmonary blood vessels and improve blood circulation in the lungs (39, 48). At high concentrations, this effect is inhibited (49, 50). Zhang et al. (51) reported that coicis oil can inhibit blood vessel formation in liver tumor tissues, thereby reducing the nutrient supply, inhibiting cancer cell proliferation and tumor growth, and improving the body’s immune response against tumors. Kanglaite injection can affect the human pancreatic cancer cell cycle, regulate gene expression, and has analgesic effects (52, 53). Kanglaite can prevent cells in the G2/M phase of the cell cycle from proceeding to the next phase, reduce the number of cells entering the G_0_ and G_1_ phases, and decrease the percentage of cells in the S phase, thereby reducing mitosis and inhibiting tumor cell proliferation. It has been widely used as an adjuvant treatment for breast, liver, lung, pancreatic, and nasopharyngeal cancers (39, 54–62) (Figure 3). Zhang et al. (51) used the MTT method to determine the anti-tumor activity of extracted coix seed oil (CSO) and found that it has inhibitory effects on colon cancer cells (SW480), human breast cancer cells (MCF7), human liver cancer cells (SMMC7721), human lung cancer cells (A549), and Myeloid leukemia cells (HL-60). Among them, the inhibition rates of these tumor cells were 95.52, 85.02, 91.83, 92.54, and 72.89%, respectively. Coix seed oil significantly inhibited the proliferation, migration and invasion of gastric cancer SGC-7901 cells, possibly by downregulating the PRMT5-PI3K/AKT signaling pathway to inhibit the activation of various anti-apoptotic molecules, induce apoptosis and suppress invasion and metastasis (63) (Figure 3). CSO induced MMP depolarization, increased mitochondrial permeability transition pore opening, increased ROS production, and further increased mitochondrial damage. In addition, CSO down-regulated the expression of p-AKT and p-PI3K proteins, up-regulated the protein expression of cleaved caspase-9, Bax, cleaved caspase-3, and cytochrome c, and down-regulated the expression of Bcl-2 by up-regulating *PTEN* gene (64) (Figure 3). CSO may have an anti-tumor effect on mice bearing 4T1 tumor by regulating arachidonic acid metabolism, biosynthesis of unsaturated fatty acids, and mutual conversion of pentose and glucuronic acid and pyruvate metabolism (54).

Coixenolide was the first discovered component of adlay and was found to have antitumor activity; it is produced by the condensation of one molecule of 2, 3-butanediol and two molecules of unsaturated fatty acids (cis-hexadecenoic acid and trans-octadecenoic acid). Coixenolide participates in cell function and regulates cell growth. It has moisturizing and anti-aging effects on skin. It can be used in skin-rejuvenating cosmetics to enhance the skin’s anti-aging function, make the skin smooth and fine, and significantly reduce the formation of facial wrinkles. Coixenolide can inhibit the growth of cancer cells and kill them (55). Coixenolide can increase the radiosensitivity of human nasopharyngeal carcinoma cells and selectively enhance the cytotoxic effects of the chemotherapeutic drugs pingyangmycin and 5-fluorouracil (5-FU) (65). Qu et al. (66) used coixenolide to treat 60 patients with advanced nasopharyngeal carcinoma with distant metastases, and the effective treatment rate was 86.7%.

Coixol, also known as adlay amide, has a melting point of 151.5_152.5°C and is soluble in acetone-petroleum ether. It was first isolated from adlay roots by Japanese scholars (67). Since then, researcher have discovered a variety of pharmacological effects of coixol, such as sedation, anti-inflammation, inhibition of multiple synaptic reactions, cooling as an antipyretic, lowering blood sugar concentration, muscle relaxation, anticonvulsant, and antithrombotic effects (33). The content of coixol in the same plant part of adlay from different habitats differs (53). Coixol may have an anti-tumor effect on mice bearing 4T1 tumor by regulating arachidonic acid metabolism, biosynthesis of unsaturated fatty acids, and mutual conversion of pentose and glucuronic acid and pyruvate metabolism (68). Adlay’roots, stems and leaves can be used for medicinal purposes instead of harvesting during the flowering and fruiting periods (ca. July_October). Coixol exerts anti-inflammatory effects by suppressing the expression of pro-inflammatory mediators *in vitro*. The mechanism of this effect was in part related to its ability to suppress NF-κB, MAPK pathways, and NLRP3 inflammasome activation in lipopolysaccharide- induced RAW 264.7 cells (33).

### 3.4 Polyphenols

**FIGURE 4:**
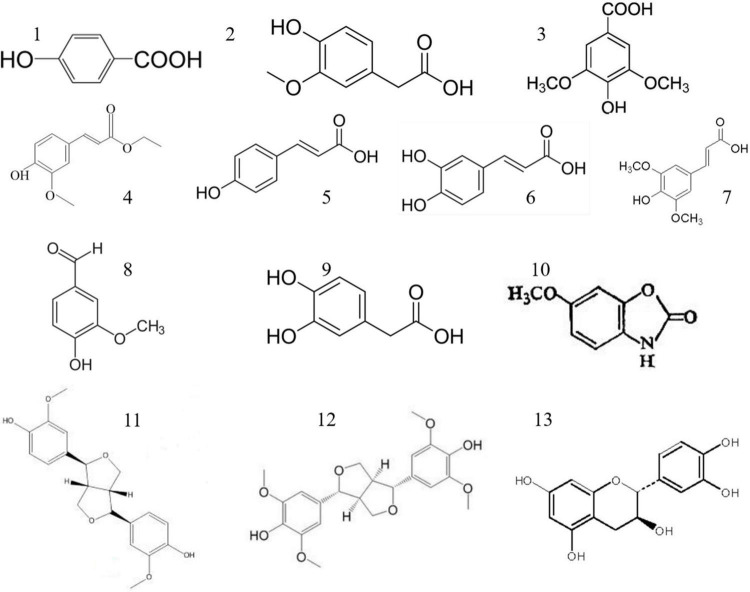
Polyphenols of adlay (*Coix lacryma-jobi* L.). (1) P-Hydroxybenzoic acid, (2) vanillic acid, (3) syringic acid, (4) ferulic acid, (5) p-coumaric acid, (6) caffeic acid, (7) erucic acid, (8) vanillin Acid, (9) 2-hydroxyphenylacetic acid, (10) coixol, (11) 4-ketopinol ester, (12) syringaresinol, (13) catechin. Photograph by JY.

In recent years, phenolic substances such as p-coumaric acid, vanillic acid, chlorogenic acid, tannic acid, catechol, and ferulic acid have been isolated from adlay husk. Thirteen free polyphenols were identified, and their structural formulae are shown in Figure 4. Flavonoids in adlay can inhibit the release of histamine and exert anti-inflammatory effects. Chang et al. (69) isolated the flavonoid eriodictyol from the chaff of adlay. Luo et al. (36) isolated 15 types of flavonoids, including ketones, dihydrochalcones, chalcones, flavanones, and isoflavones from coix bran, all of which had anti-inflammatory effects. A study done on adlay seed polyphenols mainly focused on total polyphenols content and their antioxidant activity (70). Adlay seeds contain both free and conjugated polyphenols (71). In humans, conjugated polyphenols can reach the colon and be released by *Escherichia coli*, which has certain health effects (72). Conjugated polyphenol content accounts for 45.28% of the total polyphenol content, and there is a significant correlation between the total polyphenol content of adlay seeds and total antioxidant capacity (*R*^2^ = 0.935) (44). Chen et al. (72) demonstrated that edible polyphenol extracts of coix seeds can effectively enhance the activity of T cells in the body and produce natural killer cells. Xiong et al. (73) found that a new phenolic compound from adlay protected HepG2 cells from oxidative stress by increasing antioxidant enzymes regulated by the Nrf2/ARE pathway. The phenolic compounds contained in coix bran can inhibit lipopolysaccharide stimulation of IL-6 and TNF-α secretion by mouse peritoneal macrophages, and have a clear anti-proliferative effect on tumor cells (74). The results of enzyme kinetics showed that the inhibition type of the coix phenolic acid compound on α-glucosidase was non-competitive. The inhibitory activity of total phenolic acids (1.0 mg/mL) against α-glucosidase was as high as 87.5%.

### 3.5 Vitamins and minerals

In recent years, the effects of vitamins and minerals on human health have received increasing attention (43). All parts of the adlay plant are rich in vitamins. The vitamin E and niacin contents of adlay are mainly concentrated in the seeds. Chang et al. (46) reported for the first time that the content of tocopherols in coix seed oil ranged from 0.64_1.57 mg/g, which was higher than that in olive oil (0.26_1.0 mg/g). The contents of vitamins E, C, B1, B2, B6, B12, folic acid, and niacin in the seed coat were higher than those in the seed kernel. The nutritional value of brown coix (coix in red coat) is higher than that of white coix (11). Additionally, vitamins B1, B2, and B12 were the highest in the roots, and vitamins B6 and folic acid were mainly concentrated in the leaves (75). Adlay is also rich in minerals including calcium, magnesium, phosphorus, iron, zinc, and manganese, which are necessary for human beings. The mineral contents in the roots and leaves were relatively high, whereas they were low in the seed shell and skin. Phosphorus and zinc contents were the highest in the seed kernels, reaching 1.93 and 0.15 mg/g, respectively. The contents of copper, iron and chromium in the root are the highest, being 0.01, 1.46, and 0.04 mg/g, respectively; the contents of potassium, calcium, sodium, and magnesium are the highest in the leaf, being 4.18, 15.65, 1.90, and 5.85 mg/g, respectively. Yu et al. (76) assessed mercury (Hg), lead (Pb), cadmium (Cd), arsenic (As), chromium (Cr), and 116 pesticides in coix seeds. The results showed that the pollution index and risk assessment of heavy metal and pesticide residues indicated that coix seeds were safe for consumption.

### 3.6 Other active ingredients

Adlay seeds contain a variety of sterols, including feruloyl-stigmasterol, ferulyl-brassinosterol, brassicol, α-, β-, and γ-sitosterol, and stigmasterol (77). Feruloyl- stigmasterol and ferulyl-brassinosterol can induce ovulation in golden hamsters at 200 μg/day. β-Sitosterol had anti-cancer, anti-blood cholesterol, antitussive and anti-inflammatory effects (74). Triterpenoids have good immune, hypoglycemic and hypolipidemic effects; protect liver function; and have antiviral and antitumor effects (40). Triterpenoid ring-opening compounds in coix, such as squalene and lower blood sugar, have anti-tumor activity and scavenge free radicals. Chang et al. (46) isolated two triterpenoids, friedelin, and isoarborinol, from adlay kernel. The average content of GABA in coix seeds, determined by high-performance liquid chromatography, was 20.8 mg/100 g of seeds. GABA is a non-protein amino acid that is widely present in nature and has various roles in regulating body functions, such as lowering blood pressure, improving brain function, serving as an anti-convulsant, preventing and treating epilepsy, activating kidney and liver functions, and promoting sperm fertilization (27). Amen et al. (78) isolated melanogenesis inhibitors with anti-melanoma activity from the methanol extract of yellowed adlay seedlings. In addition to the above-mentioned functional components, researchers have also isolated alkaloids, eight lactam compounds, lignin compounds, indole compounds, and spironolactones from adlay chaff. Amides and lignans were isolated from coix bran. Alcohol compounds, five kinds of benzoxazinone compounds, and adenosine were isolated from the coix roots (75). Coix seed can be used to treat a variety of cancers through different treatment pathways, fight against advanced cancer complications and cachexia, relieve symptoms such as pain, muscle, and fat loss, and improve the quality of life of patients (12). The therapeutic mechanism of coix seed for tumor in various studies in recent years are summarized in Table 3.

**TABLE 3:** Main pathway and effective components of coix seed in treatment of cancer.

Active ingredient	Corresponding disease	Pharmacological action	Pathway of action
Stigmasterol, β-sitosterol	Cervical cancer	Inhibit tumor microangiogenesis	Inhibit the secretion of vascular endothelial factor and its receptor activity
Coixan	Non-small cell lung cancer	Induce cancer cell apoptosis	Activate the endogenous mitochondrial apoptosis pathway, activate the apoptotic proteins Caspase-6 and Caspase-9, and promote cell apoptosis
Triglycerides, Coixenolide, Coixol	Pancreatic cancer	Induce cancer cell apoptosis	Down-regulate the expression of BCL-2 protein, increase the expression of Fas gene, and promote cell apoptosis; activate the PTEN tumor suppressor gene and regulate the PI3K/AKT pathway
Coixenolide	Promyelocytic leukemia cell line HL-60	Induce cancer cell apoptosis	Appear apoptotic bodies, activate the apoptotic protein Caspase-3, induce mitochondrial apoptosis and lead to cell apoptosis
Coixan; Triglycerides, Coixenolide, Coixol; Triglycerides, Coixenolide, Coixol and Erlotinib	Non-small cell lung cancer	Inhibit cancer cell metastasis	Down-regulate the expression of S100 calbindin A4; Inhibit the enzyme activity of MMPs and enhance the activity of TIMPs; Inhibit the expression of proliferation protein, invasion protein, matrix protein and JAK2/STAT3 signaling pathway
Triglycerides, Coixenolide, Coixol	Laryngeal cancer cell line Hep-2	Inhibit cancer cell metastasis	Inhibit the expression of invasion and transfer factors
Stigmasterol, β-sitosterol	Uterine fibroids	Inhibit cancer cell proliferation	Reduce the expression of vascular endothelial factor and inhibit the secretion of female sex hormones; Inhibit the sex hormone diethylstilbestrol/medroxyprogesterone 17 acetate-induced myometrial hyperplasia
Triglycerides, Coixenolide, Coixol	Gastric cancer cell line SCG-7901	Inhibit cancer cell proliferation	Inhibit the expression of Bcl-2 gene to block cancer cells in the G1 cycle
Coix Seed Ethanol Extract	Non-small cell lung cancer A549	Inhibit cancer cell proliferation	Inhibit the expression of cyclin A and block the proliferation of cancer cells during the G1/S transition of the cell cycle
Coix Seed Oil Extract	Lewis lung cancer C57BL/6	Anti-cachexia	Regulate NF-κB-MuRF1 and AMPK-HSL pathways, inhibit the increase of inflammatory factors and phosphorylated HSL, thereby avoiding fat and muscle loss

BCL-2, B-cell Lymphoma 2; PTEN, phosphatase and tensin homolog deleted on chromosome ten; PI3K/AKT, phosphoinositide 3-kinase/protein kinase B; MMPs, matrix metalloproteinase; TIMPs, tissue inhibitor of metalloproteinases; JAK2/STAT3, just another kinase2/signal transducer and activator of transcription 3; NF-κB-MuRF1, nuclear factor of kappa B/muscle ring finger protein1; AMPK-HSL, adenylate activated protein kinase-hormone sensitive lipase (13, 30, 36, 45, 47, 48, 51, 55, 56, 59, 65, 66, 69, 72).

## 4 Genome and germplasm resources genetic improvement of adlay

The availability of high-quality genome sequences and population genomics resources would greatly promote research on adlay and its comparison with other important cereal crops, especially sorghum and maize, as well as future genome-assisted breeding of adlay.

### 4.1 Gene and genome of adlay

Adlay transcription factors and functional genes were cloned and identified successively. Vettore et al. (79) isolated the homologous gene *Opaque 2* of maize from adlay. This gene encodes a basic domain-leucine zipper (bZIP),DNA-binding factor. Dante et al. (80) cloned the *DapA* gene from adlay. The gene contains two introns and encodes dihydrodipicolinate synthase (DHPS) composed of 326 amino acids, which is similar to corn DHPS (up to 95%). Yoza et al. (81) cloned the cysteinase inhibitor gene (*cystatin*) from the cDNA library constructed from mature coix seeds. Its cDNA was 757 bp long and encoded 135 amino acids. Wei et al. (82) cloned 3-ketoacyl-CoA synthase (KCS), in which the full-length KCS gene was 1,548 bp encoding 515 amino acids, from cDNA of *Coix lachrymal-Jobi* L. Gene expression analysis showed that there were significant differences in β-Ketoacyl CoA synthetase genes (KCS) in adlay isolates with different fat contents. Hachiken et al. (83) used PCR to isolate the full-length coding sequence of the *waxy* gene from non-waxy coix varieties, which is a key gene in the amylose synthesis pathway. Comparing the full-length gene sequences of two non-waxy varieties and three waxy varieties, it was found that a 275 bp fragment was missing in the coding region of the gene in waxy varieties. PCR analysis revealed that this deletion is common in coix varieties in Japan and Korea. Compared to *Oryza sativa*, *Sorghum bicolor*, and other field crops, research on coix genomics and molecular biology is limited and there is currently no reference genome. Ottoboni et al. (84) cloned the adlay α*-coixin* gene using map-based cloning. The full-length cDNA sequence was 798 bp and encoded 265 amino acids. The α-coixin protein consists of an N-terminus containing a signal peptide, ten highly conserved tandem repeats of 15_20 amino acids flanked by polyglutamine domains, and a short C-terminus. Overexpression of this gene revealed that the content of lysine in coix gliadin was higher, almost 2.5 times that of zein, and the tryptophan content was also significantly higher than that in zein. To date, few draft genomes and *de novo* sequencing have been performed to discover genes responsible for nutritional traits and their molecular mechanisms, which are insufficient for the efficient utilization of existing germplasms for molecular breeding of adlay.

The discovery and utilization of functional genes with higher medicinal ingredients and protein-level traits would help to breed new elite coix cultivars. Liu et al. (16) reported a whole genome draft of adlay, with a size of approximately 1.619 Gb, of which 75.39% were repetitive sequences, and a total of 39,629 protein-coding genes were annotated. Liu et al. constructed a genetic map of adlay and located some important traits, such as the strength of the adlay husk. Anatomical and transcriptomic analyses revealed that the soft shell of adlay is formed by inhibition of cell division and wall biogenesis. They also revealed that seed shell stress resistance is controlled by two major quantitative trait loci QTLs that are related to shell thickness and color, respectively. Guo et al. (1) drew a draft genome of the forage type Daheishan adlay *de novo*, and through comparative genome analysis adlay and sorghum were found to have the closest relationship. Evidence from transcriptome sequencing indicates that the expression of genes involved in cell division and cell wall synthesis is suppressed in cartons. Population phenotype correlation analysis and backcross population background clearance analysis both indicated that Ccph1 controlled seed shell thinning, Ccph2 controlled seed shell color whitening, and white shells had more accumulation of mineral elements than black shells, which may have made the shells more brittle and crumblier. Genomics combined with population maps, genetic maps, and transcriptome databases provides a powerful platform for adlay evolution and functional genomics studies and will facilitate molecular breeding of this important crop. Kang et al. (17) generated a draft genome of *C. lacryma-jobi* var. ma-yuen Korean cultivar “Johyun” *via* the *de novo* assembly method, using PacBio and Illumina sequencing data. A total of 3,362 scaffold sequences were assembled with a length of 1.28 Gb, accounting for 82.1% of the estimated genome size (1.56 Gb). Genome integrity was confirmed by the presence of 91.4% BUSCO angiosperm genes and mapping ratio of 98.3% Illumina paired-end reads. They found that approximately 77.0% of the genome is occupied by repetitive sequences, most of which are Gypsy- and Copia-type retrotransposons. Evidence-based genome annotation predicted 39,574 protein-coding genes, 85.5% of which were functionally annotated. In recent years, the development of molecular biology technology, especially the cost reduction and wide application of high-tech technologies such as deep sequencing and proteomics, has provided rare opportunities for systematic research on adlay.

### 4.2 Germplasm resources genetic improvement of adlay

The diversity of coix germplasm resources is the basis for variety improvement and selection of new varieties. In research, it can accurately and quickly identify and evaluate germplasm resources, which can speed up breeding success. Wang et al. (71) cut the immature inflorescence of adlay into small pieces of approximately 1 cm and cultured them *in vitro* on N6 medium containing 1_2 mg/L 2, 4-D, and 3_5% sucrose to obtain calli. Then the embryogenic callus was transferred to MS medium supplemented with 0.5 mg/L KT and 0.01 mg/L NAA to obtain adlay (2*n* = 20) regeneration plants. Li et al. (27) selected the base section of seedling leaves as explants and obtained good-growing embryogenic calli on N6 medium containing high concentrations of proline and low concentrations of 2,4-D. Then, regenerated shoots were obtained on MS medium containing 6-BA, KT, and GA3. Zhang et al. (51) used the aerial roots, seed embryos, and tender stems of the dwarf and early maturing coix variety “Qiandong No. 1” as materials to induce and differentiate embryogenic calli, and cultivate detoxified adlay regenerated plants. Zhang et al. (51) believed that aerial roots were the most suitable explants for inducing embryo calli and embryoid bodies and are superior to mature embryos and tender stems. KT is the main factor influencing the differentiation of adlay buds and t generation of detoxified seedlings.

The use of molecular markers to conduct genetic diversity analysis of adlay germplasm resources and analysis of the genetic relationship between adlay and other gramineous crops has been comprehensively studied, which can provide important references for the collection, evaluation, utilization, and protection of adlay germplasms (85). At present, the variety of adlay cultivated in production is single, there is a lack of new high-quality adlay varieties, and insufficient improvement and innovative utilization of adlay germplasm resources, and wild adlay germplasm resources are also sharply reduced. Therefore, studying the genetic diversity of adlay germplasm resources is important for the discovery and selection of excellent adlay germplasm resources. Traditional hybrid breeding methods are time-consuming and laborious, whereas genome editing technology can efficiently and quickly allow for site-directed mutations and precise breeding (86). With the continuous advancement of functional genomics research, the functions of several adlay genes have been elucidated, and specific genes can undergo site-directed mutations or modifications through genome editing technology to produce expected target traits (1, 16). The use of genome editing technology can also achieve coix site-specific modification of multiple sites, quickly forming a superposition of multiple traits. Furthermore, adlay quantitative trait loci can be edited using genome editing tools. Using genome editing tools to directly edit endogenous genes can efficiently obtain haploid induction lines. Genome editing technology can also be used as directed evolution and forward genetics screening tool to identify and screen genotypes and phenotypes from the adlay whole genome.

## 5 Summary and perspectives

Coix seeds from cultivated plants are both edible and medicinal (24). Coix seed is rich in protein, fat, amino acids, and trace elements, and has high nutritional value. It also has anti-tumor, anti-inflammatory, anti-viral, anti-bacterial, hypoglycemic, and immune-regulatory functions. With the completion of adlay genome sequencing, future, functional research and the development of coix varieties can proceed in several directions. Large-scale development and utilization of genetic resources are necessary conditions for developing nutritional traits and cultivating nutrient-rich commercial coix varieties. These needs include: (i) reliable and high-throughput screening of genetic resources for nutritional traits and biologically active compounds, (ii) multilocation testing to quantify the genotype and environmental interactions of nutritionally important traits, and (iii) strengthening the use of unexplored wild coix seeds for nutritional development. The rapid emergence of “multi-level genetic and omics development” may play a key role in supplementing the development methods for the nutritional traits of the aforementioned coix. However, compared with the main crops, the use of different omics to intervene in the nutrition development and breeding program of adlay is relatively lagging. The latest advances in plant molecular breeding, such as “genome selection,” “genome editing through CRISPR-Cas9” and “rapid breeding,” will shorten the breeding time and improve nutritional traits and yield. Making full use of multilayered omics tools in coix breeding can narrow the gap with the main crops that have been intensively bred for more than a century. In short, improving the nutritional potential of adlay plays an important role in changing modern cropping systems according to the needs of future consumers.

The functional roles of the ingredients must be exploited through products. The purpose of functional ingredient research is to make use of these ingredients (87). Initial research clarified the content and function of the ingredients, and then the functional components were developed for use (88). Although there are some gene reports in adlay, most of them remain at the level of bioinformatics or expression analysis. Actual gene function, metabolism, and regulation have not been well analyzed, and the corresponding genetic transformation and other technical means need to be improved. The main directions of future coix breeding include the following:

The first is the overall layout of the collection and preservation of coix germplasm resources, especially wild coix resources, to broaden the diversity of resources for variety cultivation, and to form a dominant germplasm resource group. The second is to integrate innovative breeding technologies such as genome-wide selection and gene editing, and to combine the ideal plant type with the future super hybrid coix molecular design model to innovate third-generation coix heterosis utilization technology. The third is to use big data such as whole genome data, germplasm phenotype data, local climate, soil, and biotic and abiotic resources, to create a technical system for intelligent design of new germplasm adaptive to the ecological region. The fourth is to be guided by industrial needs, breed new varieties of coix suitable for live broadcast, mechanized production, and environment-friendly; cultivate new varieties of high-quality coix with functions such as rich iron, zinc, and selenium that meet the needs of different groups of people. The fifth goal is to further strengthen the research and development of biological breeding and promote the industrialization of biological breeding. Based on the genome, phenotype, artificial intelligence, and big data, the target genes can be edited to produce the desired phenotypes. Thus, our aim is to predict the utilization value of stock germplasm resources at the genome-wide level, quickly aggregate excellent alleles, realize the intelligent, efficient, and targeted creation of new germplasm, and promote the industrialization of biological breeding.

## Author contributions

JR and MZ designed the review. WW, XP, and YP collected the literature and drafted the manuscript. XL, ZL, JY, and AG contributed to draw tables and plots. MZ, JR, and JC responsibility for the project, contributed to the manuscript revision. WW and JR contributed to the method development and manuscript revision. All authors contributed to the article and approved the submitted version.

## References

[1] Guo C Wang Y Yang A He J Xiao C Lv S The *Coix* genome provides insights into Panicoideae evolution and papery hull domestication. *Mol Plant.* (2020) 13:309–20. 10.1016/j.molp.2019.11.008 31778843

[2] Liu X Fan K Song WG Wang ZW. Optimization of accelerated solvent extraction of fatty acids from *Coix* seeds using chemometrics methods. *J Food Meas Charact.* (2019) 13:1773–80.

[3] Liu H Li L Zou J Zhou T Wang B Sun H *Coix* seed oil ameliorates cancer cachexia by counteracting muscle loss and fat lipolysis. *BMC Complement Alternat Med.* (2019) 19:267. 10.1186/s12906-019-2684-4 31615487 PMC6792186

[4] Liu X Zhang B Xu JH Mao DZ Yang YJ Wang ZW. Rapid determination of the crude starch content of *Coix* seed and comparing the pasting and textural properties of the starches. *Starch Starke.* (2017) 69:1600115.

[5] de Luna MDG Flores ED Cenia MCB Lu MC. Removal of copper ions from aqueous solution by adlay shell (*Coix lacryma-jobi* L.) adsorbents. *Bioresource Technol.* (2015) 192:841–4. 10.1016/j.biortech.2015.06.018 26081160

[6] Xu L Yang N Wu F Jin Z Xu X. Impact of germination on the chemical components and bioactive properties of Adlay (*Coix lachrymal-jobi* L.) water extract. *Int J Food Sci Technol.* (2018) 53:449–56.

[7] Yin H Zhong Y Xia S Hu J Nie S Xiong T Effects of fermentation with *Lactobacillus plantarum* NCU137 on nutritional, sensory and stability properties of *Coix* (*Coix lachryma-jobi* L.) seed. *Food Chem.* (2019) 12:6037. 10.1016/j.foodchem.2019.126037 31954941

[8] Yang Z Zhu X Wen A Ran J Qin L Zhu Y. *Coix* seed-based milk fermented with *Limosilactobacillus reuteri* improves lipid metabolism and gut microbiota in mice fed with a high-fat diet. *Front Nutr.* (2022) 9:921255. 10.3389/fnut.2022.921255 35903451 PMC9320324

[9] Djaja N Permadi I Witjaksono F Soewondo P Abdullah M Agustina R The effect of Job’s tears-enriched yoghurt on GLP-1, calprotectin, blood glucose levels and weight of patients with type 2 diabetes mellitus. *Med J Nutr Metab.* (2019) 12:163–71.

[10] Xu M He D Teng H Chen L Song H Huang Q. Physiological and proteomic analyses of coix seed aging during storage. *Food Chem.* (2018) 260:82–9. 10.1016/j.foodchem.2018.03.129 29699686

[11] Huang Q Xu M Zhang H He D Kong Y Chen L Transcriptome and proteome analyses of the molecular mechanisms associated with *Coix* seed nutritional quality in the process of breeding. *Food Chem.* (2019) 272:549–58. 10.1016/j.foodchem.2018.07.116 30309580

[12] Yu Q Ye G Lei Z Yang R Chen R He T An isolated compound from stems and leaves of *Coix lacryma-jobi* L. and its anticancer effect. *Food Biosci.* (2021) 42:101047.

[13] Son ES Kim SH Kim YO Lee YE Kyung SY Jeong SH *Coix lacryma-jobi var. ma-yuen* Stapf sprout extract induces cell cycle arrest and apoptosis in human cervical carcinoma cells. *BMC Complement Alternat Med.* (2019) 19:312. 10.1186/s12906-019-2725-z 31729992 PMC6858790

[14] Nishimura M Ohkawara T Kagami-Katsuyama H Sekiguchi S Taira T Tsukada M Alteration of intestinal flora by the intake of enzymatic degradation products of adlay (*Coix lachryma-jobi* L. var. ma-yuen Stapf) with improvement of skin condition. *J Funct Food.* (2014) 7:487–94.

[15] Zhang W Jia X Xu Y Xie Q Zhu M Zhang H Effects of *Coix* seed extract, *Bifidobacterium* BPL1, and their combination on the glycolipid metabolism in obese mice. *Front Nutr.* (2022) 9:939423. 10.3389/fnut.2022.939423 35923203 PMC9341295

[16] Liu H Shi J Cai Z Huang Y Lv M Du H Evolution and domestication footprints uncovered from the genomes of *Coix*. *Mol Plant.* (2020) 13:295–308. 10.1016/j.molp.2019.11.009 31778842

[17] Kang SH Kim B Choi BS Lee HO Kim NH Lee SJ Genome assembly and annotation of soft-shelled Adlay (*Coix lacryma-jobi* variety ma-yuen), a cereal and medicinal crop in the Poaceae family. *Front Plant Sci.* (2020) 11:630. 10.3389/fpls.2020.00630 32528499 PMC7247446

[18] Huang DW Chung CP Kuo YH Lin YL Chiang W. Identification of compounds in Adlay (*Coix lachryma-jobi* L. var. ma-yuen Stapf) seed hull extracts that inhibit lipopolysaccharide-induced inflammation in RAW 264.7 macrophages. *J Agric Food Chem.* (2009) 57:10651–7. 10.1021/jf9028514 19886607

[19] Zhu R Xu X Shan Q Wang K Cao G Wu X. Determination of differentiating markers in coicis semen from multi-sources based on structural similarity classification coupled with UPCC-Xevo G2-XS QTOF. *Front Pharmacol.* (2020) 11:549181. 10.3389/fphar.2020.549181 33178013 PMC7596418

[20] Pandey A Tomer AK Bhandari DC Pareek SK. Towards collection of wild relatives of crop plants in India. *Genet Resour Crop Evol.* (2008) 55:187–202. 10.18699/VJ20.622 33659813 PMC7907825

[21] Fu Y Yang C Meng Q Liu F Shen G Zhou M Genetic diversity and structure of *Coix lacryma-jobi* L. from its world secondary diversity center, Southwest China. *Int J Genomics.* (2019) 2019:9815697. 10.1155/2019/9815697 30805354 PMC6360581

[22] Ruan JJ Weng WF Yan J Zhou YX Chen H Ren MJ *Coix lacryma-jobi* chymotrypsin inhibitor displays antifungal activity. *Pestic Biochem Phys.* (2019) 160:49–57. 10.1016/j.pestbp.2019.06.016 31519257

[23] Xuan L Xi X Xu Z Xie H Zhu Y Cheng Z Genetic differences and variation in polysaccharide antioxidant activity found in germplasm resources for Job’s tears (*Coix lacryma-jobi* L.). *Botany.* (2020) 98:651–60.

[24] Shen G Girdthai T Liu ZY Fu YH Meng QY Liu FZ. Principal component and morphological diversity analysis of Job‘s-tears (*Coix lacryma-jobi* L.). *Chil J Agric Res.* (2019) 79:131–43.

[25] Laxmisha KM Semwal DP Gupta V Katral A Bisht IS Mehta PS Nutritional profiling and GIS-based grid mapping of Job’s tears (*Coix lacryma-jobi* L.) germplasm. *Appl Food Res*. (2022) 2:100169.

[26] Liu X Yang YJ Wang ZW. Structure characteristics of *Coix* seeds prolamins and physicochemical and mechanical properties of their films. *J Cereal Sci.* (2018) 79:233–9.

[27] Li B Qiao L Li L Zhang Y Li K Wang L Novel antihypertensive peptides derived from Adlay (*Coix larchryma-jobi* L. var. ma-yuen Stapf) glutelin. *Molecules.* (2017) 22:123. 10.3390/molecules22040534 28346383 PMC6154315

[28] Liu X Zhang X Rong YZ Wu JH Yang YJ Wang ZW. Rapid determination of fat, protein and amino acid content in coix seed using near-infrared spectroscopy technique. *Food Anal Method.* (2015) 8:334–42.

[29] Luo X Li H Jiang D Meng J Zhang F Xu Q Analysis of fungi on *Coix* (*Coix lacryma-jobi*) seed and the effect of its aqueous extract on the growth of *Aspergillus flavus*. *J Food Protect.* (2019) 82:1775–82. 10.4315/0362-028X.JFP-19-019 31545107

[30] Lin PH Shih CK Yen YT Chiang W Hsia SM. Adlay (*Coix lachryma-jobi* L. Var. Ma-yuen Stapf.) hull extract and active compounds inhibit proliferation of primary human leiomyoma cells and protect against sexual hormone-induced mice smooth muscle hyperproliferation. *Molecules.* (2019) 24:1556. 10.3390/molecules24081556 31010220 PMC6514562

[31] Hong SS Choi CW Choi YH Oh JS. Coixlachryside A: a new lignan glycoside from the roots of *Coix lacryma-jobi* L. var. ma-yuen Stapf. *Phytochem Lett.* (2016) 17:152–7.

[32] Hu X Xu F Li J Li J Mo C Zhao M Ultrasonic-assisted extraction of polysaccharides from *Coix* seeds: optimization, purification, and in vitro digestibility. *Food Chem.* (2022) 374:131636. 10.1016/j.foodchem.2021.131636 34875432

[33] Zhang C Zhang W Shi R Tang B Xie S. *Coix lachryma-jobi* extract ameliorates inflammation and oxidative stress in a complete Freund’s adjuvant-induced rheumatoid arthritis model. *Pharm Biol.* (2019) 57:792–8. 10.1080/13880209.2019.1687526 31747811 PMC6882456

[34] Li Y Tian X Li S Chang L Sun P Lu Y Total polysaccharides of Adlay bran (*Coix lachryma-jobi* L.) improve TNF-α induced epithelial barrier dysfunction in Caco-2 cells *via* inhibition of the inflammatory response. *Food Funct.* (2019) 10:2906–13. 10.1039/c9fo00590k 31070650

[35] Seo GD. Development of functional enzymes and diet supplement products through microbial cereal grain cultivation technology. *Res J Pharm Technol.* (2019) 12:5042–6.

[36] Luo C Wang X An C Hwang CF Miao W Yang L Molecular inhibition mechanisms of cell migration and invasion by *Coix* polysaccharides in A549 NSCLC cells *via* targeting S100A4. *Mol Med Rep.* (2017) 15:309–16. 10.3892/mmr.2016.5985 27922683

[37] Chen J Chen Y Ge H Wu C Pang J Miao S. Multi-scale structure, pasting and digestibility of adlay (*Coix lachryma-jobi* L.) seed starch. *Food Hydrocoll.* (2019) 89:885–91.

[38] Xia T Liu CS Hu YN Luo ZY Chen FL Yuan LX *Coix* seed polysaccharides alleviate type 2 diabetes mellitus *via* gut microbiota-derived short-chain fatty acids activation of IGF1/PI3K/AKT signaling. *Food Res Int.* (2021) 150:110717. 10.1016/j.foodres.2021.110717 34865748

[39] Liu S Li F Zhang X. Structural modulation of gut microbiota reveals *Coix* seed contributes to weight loss in mice. *Appl Microbiol Biotechnol.* (2019) 103:5311–21. 10.1007/s00253-019-09786-z 30993386

[40] Kang JH Song KB. Characterization of Job’s tears (*Coix lachryma-jobi* L.) starch films incorporated with clove bud essential oil and their antioxidant effects on pork belly during storage. *LWT Food Sci Technol.* (2019) 111:711–8.

[41] Kim EJ Kim HR Choi SJ Park CS Moon TW. Low digestion property of amylosucrase-modified waxy adlay starch. *Food Sci Biotechnol.* (2016) 25:457–60. 10.1007/s10068-016-0063-1 30263291 PMC6049214

[42] Manosroi J Khositsuntiwong N Manosroi A. Biological activities of fructo- oligosaccharide (FOS)-containing *Coix lacryma-jobi* Linn. extract. *J Food Sci Technol.* (2014) 51:341–6. 24493893 10.1007/s13197-011-0498-6 PMC3907639

[43] Liu X Rong YZ Zhang X Mao DZ Yang YJ Wang ZW. Rapid determination of total dietary fiber and minerals in *Coix* seed by near-infrared spectroscopy technology based on variable selection methods. *Food Anal Method.* (2015) 8:1607–17.

[44] Huang DW Kuo YH Lin FY Lin YL Chiang W. Effect of Adlay (*Coix lachrym a-jobi* L. var. ma-yuen Stapf) testa and its phenolic components on Cu2+-treated low-density lipoprotein (LDL) oxidation and lipopolysaccharide (LPS)-induced inflammation in RAW 264.7 macrophages. *J Agric Food Chem.* (2009) 57:2259–66. 10.1021/jf803255p 19243096

[45] Bai C Zheng J Zhao L Chen L Xiong H McClements DJ. Development of oral delivery systems with enhanced antioxidant and anticancer activity: *Coix* seed oil and β-carotene coloaded liposomes. *J Agric Food Chem.* (2018) 67:406–14. 10.1021/acs.jafc.8b04879 30566345

[46] Chang WC Hu YT Huang Q Hsieh SC Ting Y. Development of a topical applied functional food formulation: Adlay bran oil nanoemulgel. *LWT Food Sci Technol.* (2020) 117:108619.

[47] Sainakham M Manosroi A Abe M Manosroi W Manosroi J. Potent in vivo anticancer activity and stability of liposomes encapsulated with semi-purified Job‘s tear (*Coix lacryma-jobi* Linn.) extracts on human colon adenocarcinoma (HT-29) xenografted mice. *Drug Deliv.* (2016) 23:3399–407. 10.1080/10717544.2016.1189464 27169326

[48] Chen Y Qu D Fu R Guo M Qin Y Guo J A Tf-modified tripterine-loaded coix seed oil microemulsion enhances anti-cervical cancer treatment. *Int J Nanomed.* (2018) 13:7275. 10.2147/IJN.S182475 30510417 PMC6231517

[49] Guo J Yuan C Huang M Liu Y Chen Y Liu C *Ganoderma lucidum*-derived polysaccharide enhances *Coix* oil-based microemulsion on stability and lung cancer-targeted therapy. *Drug Deliv.* (2018) 25:1802–10. 10.1080/10717544.2018.1516006 30343605 PMC6201799

[50] Manosroi J Chankhampan C Kitdamrongtham W Manosroi W Manosroi A. Potent in vitro Anti-mouth Cancer (KB) and immunostimulating activities of the job’s tears (*Coix lachrym a-jobi* Linn.) seed semi-purified extract cocktails containing linoleic acid. *J Oleo Sci.* (2019) 68:351–9. 10.5650/jos.ess18255 30930371

[51] Zhang P Meng X Tang X Ren L Liang J. The effect of a coix seed oil injection on cancer pain relief. *Support Care Cancer.* (2019) 27:461–5. 10.1007/s00520-018-4313-z 29971522

[52] Chen C Ai QD Wei YH. Kanglaite enhances the efficacy of cisplatin in suppression of hepatocellular carcinoma *via* inhibiting CKLF1 mediated NF-κB pathway and regulating transporter mediated drug efflux. *J Ethnopharmacol.* (2021) 264:113388. 10.1016/j.jep.2020.113388 32918990

[53] Zheng J Bai C Peng H Zhao L Xiong H. Thermosensitive magnetoliposome-Novel carrier for targeted delivery and triggered release of *Coix* seed oil. *J Magn Magn Mater.* (2020) 497:166012.

[54] Fang T Jiang Y Chen L Huang L Tian X Zhou Y *Coix* seed oil exerts an anti-triple-negative breast cancer effect by disrupting miR-205/S1PR1 axis. *Front Pharmacol.* (2020) 11:529962. 10.3389/fphar.2020.529962 33101013 PMC7556270

[55] Guo M Qu D Qin Y Chen Y Liu Y Huang M Transferrin-functionalized microemulsions coloaded with *Coix* seed oil and tripterine deeply penetrate to improve cervical cancer therapy. *Mol Pharm.* (2019) 16:4826–35. 10.1021/acs.molpharmaceut.9b00717 31663764

[56] Manosroi A Sainakham M Chankhampan C Manosroi W Manosroi J. In vitro anti-cancer activities of Job‘s tears (*Coix lachryma-jobi* Linn.) extracts on human colon adenocarcinoma. *Saudi J Biol Sci.* (2016) 23:248–56. 10.1016/j.sjbs.2015.03.008 26981007 PMC4778515

[57] Ni C Li B Ding Y Wu Y Wang Q Wang J Anti-cancer properties of *Coix* seed oil against HT-29 colon cells through regulation of the PI3K/AKT signaling pathway. *Foods.* (2021) 10:2833. 10.3390/foods10112833 34829119 PMC8621869

[58] Son E Kim Y Park C Park K Jeong S Park J *Coix lacryma-jobi var. ma-yuen* Stapf sprout extract has anti-metastatic activity in colon cancer cells in vitro. *BMC Complement Alternat Med.* (2017) 17:486. 10.1186/s12906-017-1990-y 29110726 PMC5674821

[59] Wang D Yang C Wang Z Yang Y Li D Ding X Norcantharidin combined with *Coix* seed oil synergistically induces apoptosis and inhibits hepatocellular carcinoma growth by downregulating regulatory T cells accumulation. *Sci Rep.* (2017) 7:9373. 10.1038/s41598-017-09668-2 28839202 PMC5571147

[60] Wen J Yang T Wang J Ma X Tong Y Zhao Y. Kanglaite injection combined with chemotherapy versus chemotherapy alone for the improvement of clinical efficacy and immune function in patients with advanced non-small-cell lung cancer: a systematic review and meta-analysis. *Evid Based Complement Alternat.* (2020) 2020:8586596. 10.1155/2020/8586596 32047528 PMC7007744

[61] Xi XJ Zhu YG Tong YP Yang XL Tang NN Ma SM Assessment of the genetic diversity of different job’s tears (*Coix lacryma-jobi* L.) accessions and the active composition and anticancer effect of its seed oil. *PLoS One.* (2016) 11:e0153269. 10.1371/journal.pone.0153269 27070310 PMC4829220

[62] Zhan YP Huang XE Cao J Lu YY Wu XY Liu J Clinical safety and efficacy of Kanglaite^®^ (*Coix* seed oil) injection combined with chemotherapy in treating patients with gastric cancer. *Asian Pac J Cancer P.* (2012) 13:5319–21. 10.7314/apjcp.2012.13.10.5319 23244156

[63] Chen XY Liao DC Yu YT Wei CM Xuan LY Li S *Coix* seed oil prolongs lifespan and enhances stress resistance in *Caenorhabditis elegans*. *Biogerontology.* (2020) 21:245–56. 10.1007/s10522-020-09857-z 31960183

[64] Yang J Liu Y Lu S Sun X Yin Y Wang K *Coix* seed oil regulates mitochondrial functional damage to induce apoptosis of human pancreatic cancer cells *via* the PTEN/PI3K/AKT signaling pathway. *Mol Biol Rep.* (2022) 49:5897–909. 10.1007/s11033-022-07371-8 35543827

[65] Qian Y Xiong Y Feng D Wu Y Zhang X Chen L *Coix* seed extract enhances the anti-pancreatic cancer efficacy of gemcitabine through regulating ABCB1-and ABCG2-mediated drug efflux: a bioluminescent pharmacokinetic and pharmacodynamic study. *Int J Mol Sci.* (2019) 20:5250. 10.3390/ijms20215250 31652737 PMC6862065

[66] Qu D Liu M Huang M Wang L Chen Y Liu C Octanoyl galactose ester-modified microemulsion system self-assembled by *Coix* seed components to enhance tumor targeting and hepatoma therapy. *Int J Nanomed.* (2017) 12:2045. 10.2147/IJN.S125293 28352174 PMC5358984

[67] Koyama T Yamato M. Constituents of the root of *Coix lacryma-jobi*. *J Pharmacol Soc Jpn.* (1955) 75:699–701.

[68] Meng X Huang B Zhou L He Y Chen Q Yuan Y Construction of a *Coix* BAC library and isolation of the 22 kDa α-coixin gene cluster. *Genome.* (2010) 53:667–74. 10.1139/g10-045 20924416

[69] Chang CC Huang LH Chiang W Hsia SM. Hexane fraction of Adlay (*Coix lachryma -jobi* L.) testa ethanolic extract inhibits human uterine sarcoma cancer cells growth and chemosensitizes human uterine sarcoma cells to doxorubicin. *Phytomedicine.* (2018) 47:69–80. 10.1016/j.phymed.2018.03.056 30166110

[70] Wang L Chen C Su A Zhang Y Yuan J Ju X. Structural characterization of phenolic compounds and antioxidant activity of the phenolic-rich fraction from defatted Adlay (*Coix lachryma-jobi* L. var. ma-yuen Stapf) seed meal. *Food Chem.* (2016) 196:509–17. 10.1016/j.foodchem.2015.09.083 26593521

[71] Wang L Sun J Yi Q Wang X Ju X. Protective effect of polyphenols extracts of Adlay (*Coix lachryma-jobi* L. var. ma-yuen Stapf) on hypercholesterolemia-induced oxidative stress in rats. *Molecules.* (2012) 17:8886–97. 10.3390/molecules17088886 22836208 PMC6268808

[72] Chen C Zhang Y Gao Y Xu Q Ju X Wang L. Identification and anti-tumour activities of phenolic compounds isolated from defatted adlay (*Coix lachryma -jobi* L. var. ma-yuen Stapf) seed meal. *J Funct Foods.* (2016) 26:394–405.

[73] Xiong W Li Y Yao Y Xu Q Wang L. Antioxidant mechanism of a newly found phenolic compound from adlay (NDPS) in HepG2 cells *via* Nrf2 signalling. *Food Chem.* (2022) 378:132034. 10.1016/j.foodchem.2021.132034 35026486

[74] Chen HJ Lo YC Chiang W. Inhibitory effects of adlay bran (*Coix lachryma-jobi* L. var. ma-yuen Stapf) on chemical mediator release and cytokine production in rat basophilic leukemia cells. *J Ethnopharmacol.* (2012) 141:119–27. 10.1016/j.jep.2012.02.009 22353428

[75] Zhu F. *Coix*: chemical composition and health effects. *Trends Food Sci Technol.* (2017) 61:160–75.

[76] Yu J Wang X Yao X Wu X. Safety evaluation of heavy metal contamination and pesticide residues in *Coix* seeds in Guizhou province, China. *Foods.* (2022) 11:2286. 10.3390/foods11152286 35954054 PMC9367953

[77] Hou JJ Cao CM Xu YW Yao S Cai LY Long HL Exploring lipid markers of the quality of *Coix* seeds with different geographical origins using supercritical fluid chromatography mass spectrometry and chemometrics. *Phytomedicine.* (2018) 45:1–7. 10.1016/j.phymed.2018.03.010 29576266

[78] Amen Y Arung ET Afifi MS Halim AF Ashour A Fujimoto R Melanogenesis inhibitors from *Coix lacryma-jobi* seeds in B16-F10 melanoma cells. *Nat Prod Res.* (2017) 31:2712–8. 10.1080/14786419.2017.1292270 28278663

[79] Vettore AL Yunes JA Cord Neto G da Silva MJ Arruda P Leite A. The molecular and functional characterization of an Opaque2 homologue gene from *Coix* and a new classification of plant bZIP proteins. *Plant Mol Biol.* (1998) 36:249–63. 10.1023/a:1005995806897 9484437

[80] Dante RA Neto GC Leite A Yunes JA Arruda P. The DapA gene encoding the lysine biosynthetic enzyme dihydrodipicolinate synthase from *Coix lacryma-jobi*: cloning, characterization, and expression analysis. *Plant Mol Biol.* (1999) 41:551–61. 10.1023/a:1006367116073 10608664

[81] Yoza K Sumiko Nakamura S Yaguchi M Haraguchi K Ken-Ichi Ohtsubo K. Molecular cloning and functional expression of cDNA encoding a cysteine proteinase inhibitor, cystatin, from job’s tears (*Coix lacryma-jobi* L. var. Ma-yuen Stapf). *Biosci Biotechnol Biochem.* (2002) 66:2287–91. 10.1271/bbb.66.2287 12450152

[82] Wei XY Li Y Guo J Wang YN Huang LQ. Cloning and bioinformatic analysis of the 3-ketoacyl-CoA synthase gene in *Coix lacryma-jobi* L. *Acta Pharm Sin B.* (2021) 12:610–7.

[83] Hachiken T Masunaga Y Ishii Y Ohta T Ichitani K Fukunaga K. Deletion commonly found in Waxy gene of Japanese and Korean cultivars of job‘s tears (*Coix lacryma-jobi* L.). *Mol Breed.* (2012) 30:1747–56.

[84] Ottoboni LM Leite A Yunes JA Targon ML de Souza Filho GA Arruda P. Sequence analysis of 22 kDa-like alpha-coixin genes and their comparison with homologous Zein and Kafirin genes reveals highly conserved protein structure and regulatory elements. *Plant Mol Biol.* (1993) 21:765–78. 10.1007/BF00027110 8467075

[85] Dias SM Siqueira SF Lejeune B de Souza AP. Identification and characterization of the trnS/pseudo-tRNA/nad3/rps12 gene cluster from *Coix lacryma-jobi* L: organization, transcription and RNA editing. *Plant Sci.* (2000) 158:97–105. 10.1016/s0168-9452(00)00308-3 10996249

[86] Moreau RA Singh V Hicks KB. Comparison of oil and phytosterol levels in germplasm accessions of corn, teosinte, and job’s tears. *J Agric Food Chem.* (2001) 49:3793–5. 10.1021/jf010280h 11513668

[87] Xu L Yang N Wu F Jin Z Xu X. Effect of acid pretreatment on the physicochemical and antioxidant properties of germinated Adlay (*Coix lachrymal-jobi* L.). *J Food Process Preserv.* (2018) 42:e13511.

[88] Xu Y Zhu X Ma X Xiong H Zeng Z Peng H Enzymatic production of trans-free shortening from *Coix* seed oil, fully hydrogenated palm oil and *Cinnamomum camphora* seed oil. *Food Biosci.* (2018) 22:1–8.

